# A diagnostic void in the Congo Basin: proposing a zoonotic orthopoxvirus as the cause of a hemorrhagic fever outbreak and a call for equitable health security

**DOI:** 10.1186/s40249-025-01386-6

**Published:** 2025-11-21

**Authors:** Tshibambe Nathanael Tshimbombu

**Affiliations:** Department of Neurology, Barrow Neurological Institute, St. Joseph’s Hospital and Medical Center, 350 W. Thomas Rd., Phoenix, AZ 85013 USA

**Keywords:** Disease X, Orthopoxvirus, Zoonosis, Democratic Republic of Congo, Poverty, One health, Health equity

## Abstract

**Background:**

In February 2025, a fatal outbreak of a hemorrhagic fever-like illness emerged in the Basankusu Health Zone of the Democratic Republic of Congo (DRC), a region where the burdens of poverty and infectious disease intersect. Initial field diagnostics for common filoviruses like Ebola and Marburg returned negative, creating a critical diagnostic void and confronting local health systems with a potential “Disease X.” This opinion piece analyzes the outbreak’s unique clinical and ecological context to advance a specific, actionable hypothesis.

**Main body:**

We argue that the presenting clinical syndrome, particularly the unusual combination of hemorrhagic signs with intractable hiccups and dysphagia, is highly consistent with a fulminant zoonotic orthopoxvirus infection. We hypothesize that this spillover event is directly linked to the socio-ecological pressures of poverty, including reliance on bushmeat for protein and accelerated deforestation for subsistence agriculture and charcoal production, which increase human-wildlife contact. Framing the outbreak through this lens shifts the public health paradigm from confronting a complete unknown to managing a new variant of a known threat. This perspective underscores that the poverty-driven exploitation of ecosystems is a primary engine of novel epidemics.

**Conclusions:**

The definitive etiology of the Basankusu outbreak remains unresolved, but the clinical and ecological evidence points toward a potential zoonotic origin consistent with known patterns of pathogen emergence in the Congo Basin. In a setting constrained by limited diagnostic capacity, such evidence-informed approach provides a pragmatic framework for immediate public health action—guiding the deployment of targeted diagnostics (pan-poxvirus PCR), therapeutics (tecovirimat), and vaccines (MVA-BN). Ultimately, preventing future outbreaks of diseases of poverty requires a global commitment to investing in local diagnostic capacity, sustainable development, and equitable health security within high-risk endemic regions like the Congo Basin.

**Graphic abstract:**

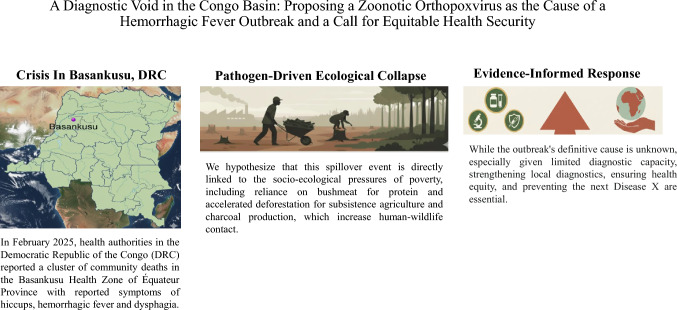

## Background

The intersection of profound poverty, ecological disruption, and microbial biodiversity has long established regions like the Congo Basin as crucibles for emerging infectious diseases [1]. For communities facing food insecurity and limited economic opportunities, the surrounding ecosystem serves as a critical, yet hazardous, resource. This dynamic creates a direct pathway for pathogen spillover from wildlife to humans, a phenomenon often catalyzed by deforestation and the bushmeat trade [2]. It is within this complex socio-ecological context that a new public health crisis emerged in early 2025.

In February 2025, health authorities in the Democratic Republic of the Congo (DRC) reported a cluster of community deaths in the Basankusu Health Zone of Équateur Province [1]. As of March 3, 2025, initial reports from the World Health Organization (WHO) and the DRC Ministry of Health indicated at least 15 cases and 12 deaths, primarily within a single-family unit, suggesting a point-source exposure followed by limited human-to-human transmission [2]. The presenting illness was described as a rapid-onset hemorrhagic fever with symptoms including high fever, headache, and severe abdominal pain [1]. Critically, field laboratory testing of initial patient samples returned negative for both Ebola virus and Marburg virus, the two most common filovirus etiologies in the region [2]. This created a “diagnostic void,” leaving public health teams confronting a potential “Disease X” scenario [3]. As of this writing, the final diagnosis for these cases remains unknown.

This diagnostic challenge is magnified by the resource-limited setting of the DRC, which is critical to understanding the call for equitable health security. The nation’s health system is chronically under-resourced, with limited laboratory infrastructure and trained personnel, especially in the rural areas where outbreaks often originate [4, 5]. Logistical barriers, including insecurity and poor infrastructure, make it exceptionally difficult to transport samples to central laboratories in a timely manner [4–6]. Furthermore, many local facilities lack consistent electricity and the basic materials required for advanced testing [4–6]. This reality of the clinical field highlights a severe gap in global preparedness and health equity, demanding attention from the global community.

Further complicating the clinical picture were reports of unusual and severe symptoms not typically dominant in filovirus infections: intractable, often hemorrhagic, hiccups and profound difficulty swallowing (dysphagia) [2]. This unique constellation of symptoms, combined with the negative filovirus results, compels a broader differential diagnosis and necessitates the formulation of an alternative, actionable hypothesis. Thus, our objective is to propose an evidence-driven, working etiology for the Basankusu outbreak to anchor a functional public health strategy in a setting defined by limited capacity. Such an inference, grounded in empirical plausibility, empowers the timely and targeted deployment of diagnostics, therapeutics, and preventive measures. Ultimately, this crisis epitomizes the urgent global mandate to confront the ecological and socioeconomic determinants of pathogen emergence, compelling a unified international resolve to strengthen local scientific capacity and avert future poverty-driven spillovers in the world’s most vulnerable regions.

## Main text

### The orthopoxvirus hypothesis: re-examining the clinical syndrome

We propose that the clinical syndrome of Basankusu Hemorrhagic Fever (BHF) is highly consistent with a fulminant, hemorrhagic presentation of a zoonotic orthopoxvirus. The *Orthopoxvirus* genus, to which the eradicated variola virus (smallpox) and the re-emerging Mpox virus belong, is capable of causing severe systemic disease [7]. While classic poxvirus infections are known for their characteristic rash, the most severe, hemorrhagic forms of smallpox were often characterized by a minimal or atypical rash, as patients succumbed rapidly to systemic viremia, shock, and a cytokine storm before skin lesions could fully develop [8]. A novel zoonotic orthopoxvirus could readily follow a similar pathogenic course.

The unusual symptoms of hemorrhagic hiccups and dysphagia are a particularly strong clue. Intractable hiccups result from irritation of the phrenic nerve or diaphragm, while dysphagia points to involvement of the glossopharyngeal (IX) and vagus (X) cranial nerves [9]. Severe, disseminated viral infections can lead to such neuropathies through direct viral invasion or intense inflammatory responses within the brainstem and peripheral nerves [10]. While uncommon, neuropathic complications, including encephalitis, have been documented in severe poxvirus infections [11]. The clinical features of BHF, when compared to filoviruses and historical hemorrhagic smallpox, show compelling overlap with the latter, providing a powerful basis for a differential diagnosis (Table 1).

**Table 1:** Comparison of clinical features of Basankusu Hemorrhagic Fever (BHF), filovirus disease, and hemorrhagic smallpox

Feature	Basankusu Hemorrhagic Fever (BHF) [1, 2]	Ebola/Marburg virus disease [12, 13]	Hemorrhagic smallpox (historical) [8]
Key clinical signs	Prominent hemorrhagic hiccups and dysphagia	Classic hemorrhagic signs; “septic” shock	Profound hemorrhage; potential for minimal rash
Transmission route	Hypothesized zoonotic; limited human-to-human	Zoonotic; human-to-human via bodily fluids	Human-to-human via respiratory droplets, fomites
Primary diagnostic method	Hypothesized pan-poxvirus PCR; Negative RT-PCR for filoviruses	Positive RT-PCR for viral RNA; Antigen-capture ELISA	Historically: electron Microscopy. Modern: pan-poxvirus PCR
Key hematologic findings	Hypothesized severe thrombocytopenia	Thrombocytopenia; early leukopenia followed by neutrophilia	Severe thrombocytopenia; potential for leukopenia
Key biochemical findings	Unknown; potential for multi-organ failure indicators	Markedly elevated liver enzymes (AST > ALT)	Elevated liver enzymes; signs of shock
Serology (acute phase)	Unknown; cross-reactivity with other orthopoxviruses possible	IgM antibodies detectable late in acute phase; not primary diagnostic tool	IgM/Antigen detection possible if patient survives long enough
Coagulopathy	Hypothesized severe coagulopathy/DIC	Present; prolonged PT/PTT, evidence of DIC	Profound and universal; DIC

*ALT* Alanine Aminotransferase, *AST* Aspartate Aminotransferase, *DIC* Disseminated Intravascular Coagulation, *ELISA* Enzyme-Linked Immunosorbent Assay, *IgG* Immunoglobulin G, *IgM* Immunoglobulin M, *PCR* Polymerase Chain Reaction, *RT-PCR* Reverse Transcription Polymerase Chain Reaction

### The socio-ecological drivers: a perspective on poverty and pathogen emergence

This outbreak cannot be divorced from the socioeconomic realities of the region. The Équateur Province, which lies within the Congo Basin, is a hotspot of deforestation, a process driven less by large-scale industry and more by the cumulative impact of subsistence activities: slash-and-burn agriculture for survival and charcoal production as a primary source of income [14]. This poverty-driven environmental degradation is a known engine of zoonotic spillover, forcing humans and wildlife into novel, high-risk interfaces [15]. While primates are the famous reservoir for Mpox, a vast reservoir of orthopoxviruses exists in small mammals, particularly rodents and squirrels (*Funisciurus anerythrus*), which are frequently hunted for bushmeat to supplement dietary protein, providing a direct route for human exposure [16–18]. We hypothesize that the initial family cluster in Basankusu was the result of a single point-source spillover event from handling an infected animal, a direct consequence of the community’s reliance on the surrounding forest for survival. This tragic event serves as a stark reminder that global health security is inextricably linked to sustainable development and poverty alleviation.

A critical question is why the DRC, one of the countries most affected by Mpox, apparently lacks the ability for rapid orthopoxvirus testing when capacity for Ebola is maintained. This discrepancy stems from a chronic lack of investment and resources dedicated specifically to Mpox. Reports indicate a significant shortage of high-sensitivity and point-of-care rapid diagnostic tests for the disease, which hinders timely detection [6]. This underinvestment is exacerbated by systemic corruption and the embezzlement of public funds [5]. In the DRC’s health sector, corruption can manifest as the diversion of resources, theft of medical supplies, and fraudulent billing, which severely undermines the system’s capacity [5]. The large-scale misappropriation of budget funds at the policy level results in extreme resource shortages for front-line services [5]. As a result, funds allocated for health system strengthening and the procurement of essential supplies are often diverted, leaving the national system unable to independently manage endemic threats [5].

This forces a reliance on a limited number of centralized labs, but ongoing conflicts and logistical barriers severely disrupt the transport of samples from remote areas [6]. Consequently, only a minority of suspected Mpox cases are ever tested, leading to significant under-diagnosis and underreporting that obscures the true scale of outbreaks [6]. This systemic failure to build decentralized testing capacity for a known endemic threat—rooted in both logistical shortfalls and domestic mismanagement—allows the virus to spread unchecked, creating the very conditions for a public health emergency and ensuring the country remains perpetually dependent on the WHO and other international partners to respond to its health crises [19].

### An actionable framework for response in a resource-limited setting

Framing BHF as a potential orthopoxvirus outbreak is not an academic exercise; it provides an immediate, actionable framework for public health response.

1. *Diagnostics*: The diagnostic strategy must be expanded immediately. Mobile laboratories should deploy pan-poxvirus PCR assays, and patient samples should be prioritized for metagenomic next-generation sequencing (mNGS) to rapidly identify and characterize a novel viral genome [15]. At the field level, this means establishing a “hub-and-spoke” model for sample management, a strategy proven effective in past Ebola responses [20]. Local health posts would serve as “spokes,” using basic sample collection kits, with designated motorcycle couriers transporting samples to a regional or district-level “hub” laboratory equipped with a GeneXpert machine [21]. This approach bypasses significant infrastructure gaps and shortens turnaround times. Furthermore, training existing community health workers to use potential future point-of-care rapid tests and to transmit results via simple, text-message-based mobile health (mHealth) platforms would provide real-time data for response teams.
2. *Therapeutics*: If confirmed, this is not a disease without treatment. The antiviral drug tecovirimat, a potent inhibitor of orthopoxvirus egress, is held in strategic stockpiles and has proven efficacy against Mpox and other related viruses. It should be considered immediately for compassionate use in suspected and confirmed cases [22, 23]. Based on lessons from the West African Ebola epidemic, implementation requires moving beyond passive stockpiling. A small, pre-approved cache of tecovirimat should be forward-deployed to designated regional treatment centers in high-risk zones like Équateur Province. National health authorities, in collaboration with the WHO, must establish a rapid-response ethical review committee capable of approving compassionate use requests within 24–48 h, ensuring that clear clinical criteria—not bureaucratic hurdles—guide treatment decisions for patients in remote clinics.
3. *Prevention*: The third-generation MVA-BN vaccine (JYNNEOS), also stockpiled globally, is highly effective in preventing orthopoxvirus infection and disease. It could be rapidly deployed in a ring vaccination strategy to protect healthcare workers, family contacts, and the broader community, effectively containing the outbreak [24, 25]. The field-level execution of ring vaccination, a strategy honed during the eradication of smallpox and used effectively in the 2018 DRC Ebola outbreak, is paramount [26]. This involves deploying mobile vaccination commando units that, upon case confirmation, rapidly identify and vaccinate all household members, neighbors, and contacts-of-contacts within a defined geographic radius. The success of this strategy is entirely dependent on community trust. Therefore, these units must include local community health workers and respected local leaders (such as village chiefs and religious figures) who can engage in culturally appropriate dialogue, dispel rumors, and champion vaccine acceptance.
4. *Health System Strengthening and Accountability*: An emergency response alone is insufficient. Addressing the root causes of this diagnostic failure requires a parallel focus on long-term systemic change. International partners must shift from a model of perpetual emergency aid to one of sustained investment in the DRC’s sovereign public health infrastructure [5, 27]. To counter the corrosive effects of corruption, funding should be tied to specific, field-tested accountability mechanisms [5, 6]. For example, a “public expenditure tracking survey” model, which has been used in other African nations, could be implemented with the help of local civil society organizations to follow funds from the health ministry down to the clinic level [28]. Furthermore, embedding independent auditors within the procurement process for diagnostics and medicines can ensure resources are not diverted. These measures help guarantee that investments build a resilient, self-sufficient health system capable of managing its own endemic threats, rather than merely funding the next emergency response.

## Conclusions

The fatal outbreak in Basankusu demands a swift response guided by a broad differential diagnosis that considers the region’s unique clinical and ecological landscape. The unique presentation, coupled with negative tests for filoviruses, strongly suggests an alternative etiology. We propose that a novel zoonotic orthopoxvirus is the most likely causative agent, with the outbreak itself being a tragic symptom of the underlying diseases of poverty and ecological distress.

This hypothesis is not merely academic; it is an actionable framework that transforms a situation of uncertainty into one with clear, tangible next steps. The global health community must act decisively on this possibility, supporting the DRC with the diagnostic and medical tools needed to contain this outbreak and prevent further loss of life in an era of increasing zoonotic threat [29]. More broadly, this event must serve as a catalyst for investment in sustainable development and local healthcare infrastructure. True pandemic preparedness is not just about stockpiling vaccines; it is about alleviating the conditions of poverty that allow such diseases to emerge in the first place.

## Data Availability

Not applicable.

## Abbreviations

BHF: Basankusu hemorrhagic fever
DRC: Democratic Republic of Congo
mNGS: Metagenomic next-generation sequencing
WHO: World Health Organization
CHW: Community health workers
ALT: Alanine aminotransferase
AST: Aspartate aminotransferase
DIC: Disseminated intravascular coagulation
ELISA: Enzyme-linked immunosorbent assay
IgG: Immunoglobulin G
IgM: Immunoglobulin M
PCR: Polymerase chain reaction
RT-PCR: Reverse transcription polymerase chain reaction
